# 40.3 MATERNAL IMMUNE ACTIVATION AND CHRONIC HALOPERIDOL INTERACT TO INCREASE MICROGLIAL ACTIVATION IN VIVO: DO ANTIPSYCHOTICS INFLAME THE BRAIN?

**DOI:** 10.1093/schbul/sby014.166

**Published:** 2018-04-01

**Authors:** Marie-Caroline Cotel, Romana Polacek, Ewelina Lenartowicz, Sridhar Natesan, Jonathan Cooper, Anthony Vernon

**Affiliations:** Institute of Psychiatry, Psychology and Neuroscience, King’s College London

## Abstract

**Background:**

Evidence-based medicine suggests that a subset of schizophrenia is associated with an inflammatory syndrome. To fully harness the potential of novel immunomodulatory therapeutics, it is critical to first determine the impact of antipsychotics on microglial function in vivo. Evidence suggests antipsychotics are anti-inflammatory; however, this is based on in vitro models and non-clinical doses of antipsychotics in vivo. It therefore remains unknown if antipsychotics promote detrimental neuroinflammation or beneficial, homeostatic changes in the brain. To address this question, we explored the effects of chronic haloperidol treatment on microglia in a rat maternal immune activation (MIA) model, representative of schizophrenia pathology.

**Methods:**

Pregnant SD rat dams were exposed to poly (I:C) on GD15 (4 mg/kg, i.v.; n=5; POL) to induce MIA, or saline (n=5; CON) as a control. At 4 months of age, male offspring from CON and POL (n=2 per litter), were randomly allocated to treatment with either haloperidol (0.5 mg/kg/d s.c.) or vehicle for 28 days by osmotic minipumps, giving four groups: CON/vehicle; CON/haloperidol; POL/vehicle and POL/haloperidol (all n=10). After 28d treatment, animals were culled and perfused transcardially with 4% PFA. Fixed brain tissues were dissected, cryoprotected and microtome sectioned (1 in 12 series, 40 µm thick). Serial sections were stained for Iba1 as a marker of microglia using an immunoperoxidase protocol. The density and morphology (soma size) of Iba1+ microglia were then assessed in the corpus striatum (CS) and anterior cingulate cortex (ACC) using unbiased stereology. Data were analysed using 2x2 ANOVA in SPSS with main effects of prenatal, postnatal and pre x post-natal interactions.

**Results:**

There were significant main effects of prenatal exposure to POL on Iba1+ microglia density in the CS (F(1,32)=18.09; p<0.001) and the ACC (F(1,32)=5.04; p<0.05) and for Iba1+ microglia soma sizes (increased) in POL offspring in both the CS (F(1,32)=88.5; p<0.001) and ACC (F(1,32)=45.06; p<0.001). There were no main effects of postnatal treatment (vehicle or haloperidol) on Iba1+ microglia density in either the CS or ACC, but there were main effects of postnatal treatment for Iba1+ microglia soma size in both CS (F(1,32)=17.3; p<0.001) and ACC (F(1,32)=7.69; p<0.01). Strikingly, there were significant interactions between pre- and post-natal treatments for both Iba1+ density in the CS (F(1,32)=5.15; p<0.05) and PFC (F(1,32)=9.43; p<0.01) as well as soma size in the CS (F(1,32)=11.6; p<0.01) and ACC (F(1,32)=11.7; p<0.01). Post-hoc testing on this interaction confirmed a significant increase in both Iba1+ density and soma size in poly(I:C)-exposed offspring treated with haloperidol, relative to all other groups in both the CS (p<0.01) and ACC (p<0.01).

**Discussion:**

Our data suggest increased microglial density and activation in the CS and PFC of rats exposed to POL in utero. Haloperidol treatment for 28 days replicating clinically comparable dosing and pharmacokinetics did not affect microglia density in saline-exposed offspring, but increased Iba1+ soma size in both CS and ACC, also suggestive of microglial activation. Strikingly, there were significant interactions between prenatal POL exposure and post-natal haloperidol treatment, leading to significantly increased microglia density and soma size in both CS and ACC. Taken together, these preliminary data suggest adult haloperidol treatment may interact with prenatal immune activation to worsen neuroinflammation.

